# Comprehensive Morpho-Physiological Responses Underlying Salt Tolerance at Different Time Points in *Brassica napus* Seedlings

**DOI:** 10.3390/plants15040661

**Published:** 2026-02-22

**Authors:** Maria Batool, Ali Mahmoud El-Badri, Lei Zheng, Chunyun Wang, Zongkai Wang, Muhammad Ikram, Maaz Ullah, Muhammad Ikram, Muhammad Waqas, Jie Kuai, Chunyu Zhang, Jinxiong Shen, Bo Wang, Guangsheng Zhou

**Affiliations:** 1 MOA Key Laboratory of Crop Ecophysiology and Farming System in the Middle Reaches of the Yangtze River, College of Plant Science & Technology, Huazhong Agricultural University, Wuhan 430070, China; maria.batool@webmail.hzau.edu.cn (M.B.); wangbo@mail.hzau.edu.cn (B.W.); 2 Field Crops Research Institute, Agricultural Research Center (ARC), Giza 12619, Egypt; 3 Sanya Institute of Breeding and Multiplication, Hainan University, Sanya 572025, China; 4 College of Plant Science & Technology, Huazhong Agricultural University, Wuhan 430070, China

**Keywords:** rapeseed, salinity, osmotic adjustment, ion homeostasis, stress tolerance

## Abstract

Soil salinization is a major environmental hazard, hindering rapeseed development due to sodium ion (Na^+^) toxicity and ionic imbalances in plant cells. Understanding tolerance mechanisms and categorizing reliable physiochemical indicators is vital for enhancing rapeseed tolerance. Herein, we aimed to enhance knowledge about the stress-responsive mechanism of ten rapeseed varieties (C71, C88, C91, C97, C123, C136, C196, C272, C280, and C320) exposed to five NaCl concentrations (0, 150, 200, 250, and 300 mM) through determining key factors related to salt tolerance at the seedling stage. Our results showed that salt stress significantly reduced seedling growth and biomass with increasing salt stress concentration in a similar pattern in all studied varieties, especially in sensitive seedlings. Furthermore, photosynthetic pigment, osmotic solutes, and MDA showed significant variations under salt treatment versus control in all studied varieties. Based on morpho-physiochemical trait analysis of ten rapeseed varieties, C71 and C272 were selected as tolerant and sensitive varieties to study stress responses during six weeks (weekly time points) in the leaf, petiole, stem, and root of seedlings under 250 mM NaCl. Current findings demonstrated superior osmotic adjustment of C71 through higher accumulation of total soluble sugars and protein, reflected in lower MDA levels, which contributed to maintaining cellular homeostasis and membrane integrity to improve resilience under salinity versus C272. Besides, total amino acid content was enhanced in C71 versus C272 seedlings, which was attributed to stress tolerance. In different tissues of C71 and C272, Na^+^ and K^+^ levels varied with increasing growing time, reaching the maximum increment at the 6th week under salt stress conditions. Moreover, Na^+^ initially accumulates in roots and enhances the K^+^ level in tolerant seedlings; besides, K^+^ was accumulated higher in the roots of tolerant seedlings, resulting in K^+^ homeostasis, thereby improving stress tolerance. Our results can be a great reference value for rapeseed plant breeders to develop salt-tolerant cultivars.

## 1. Introduction

*Brassica napus* L. (rapeseed) is the second-largest oilseed crop worldwide, providing cooking oil for human consumption, renewable materials for energy (50–70% of European biodiesel production), and industrial applications [1,2]. Rapeseed comprises high-quality oil with higher polyunsaturated fatty acids content and palatable protein (37%) feed for livestock [3]. Canada is the largest rapeseed producer, followed by China and India, occupying a large share of oil crop land and contributing to humane food and animal feed consumption [4]. In China, rapeseed production accounts for one-fifth of global rapeseed production with 11.9 million tons, and imports rapeseed oil with 2.5 million tons from other countries [5].

Furthermore, agricultural productivity is a serious problem due to abrupt climatic variation, including abiotic stresses. Among them, salt stress is one of the critical factors affecting plant growth and crop production [6,7]. About 20% of irrigated land and ∼6% of the world’s land is affected by salinity conditions, which have seriously decreased approximately 70% of crop yield [7]. Additionally, salt stress affects osmotic and ionic imbalance, generating various oxidants and free radicals that seriously influence plant growth [8]. It significantly enhances excessive reactive oxygen species (ROS) production, disrupting the enzymatic structure, nucleic acids, and cell configuration [9]. Furthermore, salinity stress significantly depreciates the photosynthetic process by affecting the electron transport chain and oxidative damage, which destroys cellular integrity and chlorophyll structure, thus disrupting key metabolic processes in the plants [10]. A previous study reported that salt tolerance correlated with cellular ionic sequestration, tolerance mechanisms, and plant growth rate in rapeseed [11]. Moreover, root growth highly decreased due to salt-induced impairment in the length and weight of *B. juncea* plants [12,13]. In China (a densely populated country), large-scale reclamation of salt-affected soils is a critical route to enhance the arable land that improves food security and closes the projected food gap [14]. Soil improvement is an important approach for soil reclamation through lower evaporation, irrigation improvements, salinity barrier reduction, and salinization monitoring efforts that reduce the annual average of 2.27 × 10^5^ hm^−2^ in saline-alkali soils in the past decade [15].

Rapeseed is a moderately salt-tolerant crop with potential adaptability, whereas the diversity of salt tolerance existed among various rapeseed cultivars [10,16]. Salt tolerance varied at different growth stages, whereas the variation of tolerance ability is higher in dicotyledonous than monocotyledonous plants [17]. Plants respond to salinity stress through various physiochemical processes for ionic homeostasis and ion detoxification, which regulate oxidative damage to prevent ion toxicity [18]. Osmotic adjustment is one of the effective adaptive mechanisms in which halophyte plants accumulate osmolytes, including low-molecular-weight compounds (metabolites, polyols, and various sugars) [19]. It proficiently enhances the cell turgor and expansion under salt-induced osmotic stress, thereby improving stress adaptation [20]. Moreover, plants cope with salt stress conditions through the de novo synthesis of osmotic substances for osmotic adjustment and accumulate inorganic ions in the cytoplasm for ionic balance [21,22]. Excessive sodium ions (Na^+^) accumulation affects the potassium (K^+^) homeostasis (involved in various metabolic processes); therefore, Na^+^/K^+^ balance is a hallmark for avoiding ionic toxicity [23]. In addition, Na^+^ exclusion (shoot), Na^+^ extrusion (root), and Na^+^ sequestration (vacuole) are important tactics of plants to prevent excessive cytosolic Na^+^ accumulation, thereby reducing Na^+^ toxicity [24,25,26]. Additionally, rapeseed is considered one of the higher Na^+^ accumulating crops compared to corn, sorghum, wheat, millet, and soybean [27]. Under higher salt stress conditions, tolerant rapeseed plants sequester Na^+^ into vacuoles and partition it into specific tissues to prevent cytosolic toxicity. Besides, it improved antioxidant activities in the aboveground parts and transferred Na^+^ to the petiole as an additional tolerance strategy [28]. Conclusively, salt tolerance is attributed to efficient sequestration, controlled compartmentalization, cell wall retention, and tissue-specific partitioning of Na^+^ rather than cytosolic accumulation [29,30].

Excessive Na^+^ alters the cellular biochemical process, which reduces plant biomass and induces hormonal imbalance and oxidative and ionic stress, thereby hindering plant growth and development [31]. Soluble sugars and proteins, which are important osmo-protectant and signaling molecules with their primary function as ROS scavengers, are involved in various stress-responsive mechanisms and actively modulate different metabolic mechanisms at different growth stages [32,33]. Moreover, amino acids are closely related to energy and carbohydrate metabolism, which work as precursors of various multi-functional secondary metabolites and signaling molecules [34]. Proline is one of the amino acids, acts as an osmo-protectant and signaling molecule that accumulates in the cytosol, and works to stabilize and protect cellular membranes, proteinaceous enzymes, and protein configuration [35]. It modulates plant metabolic processes by upregulating membrane proteins and ROS scavengers and maintaining cell solute homeostasis under stress conditions [36]. Besides, proline improves water uptake and the antioxidant system, as well as reduces excessive ion accumulation under salt stress conditions [37].

Understanding tolerance mechanisms and categorizing reliable physiochemical indicators related to their mechanisms is vital for enhancing rapeseed tolerance and sustainable agricultural development. Moreover, it is essential to identify salt-tolerant germplasm to develop a salt tolerance breeding program. Therefore, the current investigation aimed to enhance knowledge about the stress response of morpho-physiological attributes of rapeseed by determining key factors related to salt tolerance. Seedling development, photosynthetic pigments, osmolyte accumulation, and lipid peroxidation were assessed to investigate the influences of salinity conditions in rapeseed during the seedling stage.

## 2. Materials and Methods

### 2.1. Plant Materials and Growth Conditions

A panel comprising ten promising varieties of *Brassica napus* L. (C71, C88, C91, C97, C123, C136, C196, C272, C280, and C320) was used to study the deleterious effects of salt stress on morpho-physiochemical indicators at the seedling stage in a pot experiment. Each pot was filled with seven kg of air-dried soil, and a commercial compound fertilizer (N:P:K; 15:15:15%; Yishizhuang, Yichang City, Hubei Province, China) with trace micronutrients, including B (0.02%) and Zn (0.03%), was incorporated at 1.34 g per kg of soil. Each pot contains 24 small planting holes with 3–5 uniform-sized healthy seeds from each variety in a controlled-environment growth room with regulated temperature, light, and humidity (16 h light at 13,000 lx and 26 °C ± 2 °C; 8 h dark at 21 °C ± 2 °C; relative humidity 60–65%). Seedlings were thinned to keep only the strongest in each planting hole. After one month of sowing (2nd leaf stage), five concentrations of NaCl solution (0, 150, 200, 250, and 300 mM) were applied and continued for six weeks of treatment.

### 2.2. Morphological Traits Determination

Shoot length (SL), root length (RL), shoot fresh weight (SFW), and root fresh weight (RFW) of individual seedlings were measured at different treatments of ten rapeseed varieties. Moreover, they have been measured in chosen tolerant and sensitive varieties at different time points. The seedling shoots were subjected to drying in an oven at 80 °C until constant weight to determine shoot dry weights (SDW). For root characteristics, seedling roots were carefully cleaned and then scanned using Epson Expression 1640 L (Epson America, Inc., Long Beach, CA, USA). The scanned root images were subjected to the root analysis system Wseen LA-S (Hangzhou Wseen Testing Technology Co., Ltd., Hangzhou, China) to measure root surface area and root volume per seedling [38].

### 2.3. Determination of Photosynthetic Pigments

For the determination of total chlorophyll (chl) contents in fresh leaves, 0.1 g of the sample was incubated overnight with ethanol then subjected to centrifugation. Afterward, supernatant was collected, and absorbance was noted with a spectrophotometer (Beckman Coulter Inc., Fullerton, CA, USA) at 649 and 665 nm, where chl was expressed as μg g^−1^ FW [39].

### 2.4. Determination of Total Soluble Sugar and Total Soluble Protein Contents

To determine total soluble sugar, a 0.1 g fresh sample (shoot) was thoroughly mixed with 10 mL of water and boiled for 30 min at 100 °C, followed by centrifugation. The supernatants were collected and mixed with sulfuric acid-anthrone reagent, followed by boiling for 10 min at 95–100 °C in a water bath, then cooling at room temperature. The absorption value was read at 620 nm using a UV spectrophotometer [40]. While 0.1 mL of the previous supernatants was mixed with Coomassie brilliant blue (CBB) to estimate the total soluble protein. Bovine serum albumin was used as a standard, and the absorbance value was read at 595 nm on a UV spectrophotometer (Beckman Coulter Inc., Fullerton, CA, USA) [41,42].

### 2.5. Assessment of Proline Content

Fresh shoot samples (0.1 g) were mixed with 3% aqueous sulfosalicylic acid, and the homogenate was centrifuged. Then, supernatants were mixed thoroughly with glacial acetic acid and ninhydrin reagent, boiled for 30 min, cooled, and centrifuged for 5 min at 10,000 rpm. The mixture was extracted with 4 mL toluene, followed by vortex mixing, and the absorption values were noted using a UV spectrophotometer (Beckman Coulter Inc., Fullerton, CA, USA) at 520 nm [43].

### 2.6. Determination of Total Amino Acid Content

Shoot samples (0.1 g) were extracted in phosphate buffer solution (pH 7.0). Afterward, the ninhydrin reagent was added to the supernatants with vigorous mixing. Then the mixture was subjected to heating, and the absorbance value of the samples was measured at 570 nm on a UV spectrophotometer (Beckman Coulter Inc., Fullerton, CA, USA) [44].

### 2.7. Determination of Malonaldehyde (MDA) Content

To determine MDA content, 0.1 g of fresh shoot sample was homogenized with 5 mL of 0.1% (*w*/*v*) trichloroacetic acid (TCA) and then centrifuged for 20 min. A total of 4 mL of 0.5% thiobarbituric acid (TBA) dissolved in 20% TCA solution was added to 1 mL of the collected supernatants. The reaction solution was heated for 30 min at 95 °C, then cooled and centrifuged for 15 min, and the supernatants were collected carefully. The MDA content was calculated after recording the absorbance value at 450, 532, and 600 nm by a UV spectrophotometer (Beckman Coulter Inc., Fullerton, CA, USA) [41].

### 2.8. Measurement of Sodium and Potassium Ion Accumulation in Different Tissues

Leaves, roots, stems, and petioles of each seedling were separately collected and subjected to oven drying at 80 °C for 48 h. A 0.1 g sample was digested with concentrated sulfuric acid overnight. Sample tubes were placed in a digestive furnace at 160 °C for 20 min, the temperature increased to 240 °C, and when white smoke appeared in the tube, 30% hydrogen peroxide solution was slowly added drop by drop (7–8), then left for 15 min until the solution became transparent and clear. Afterward, the solution was diluted up to 50 mL, and then the filtrate was obtained; the absorbance was measured using a flame photometer (FP6431, Shanghai Yidian Analysis Instrument Co., Ltd. Shanghai, China) [39]. Ion content was calculated using the following formula

$$Ion\;content\;(mg/gDW)=\frac{X\times V}{W}\times 1000$$

where

X: Sample concentration measured by flame spectrophotometer.

V: Sample volume (mL) at the time of measurement.

W: Measured dry weight (g).

### 2.9. Statistical Analysis

The experiment was carried out in a randomized complete design in three replications. One-way ANOVA for ten varieties in each treatment and two-way ANOVA for time course analysis have been used for statistical analysis using the Statistix 8.1 software. The differences among treatments were determined by Duncan’s Multiple Range Test (DMRT) at *p* < 0.05. The graphical presentation was carried out using GraphPad Prism 8 software.

## 3. Result

### 3.1. Variation in Seedling Growth-Related Traits Under Salt Stress

**Figure 1:**
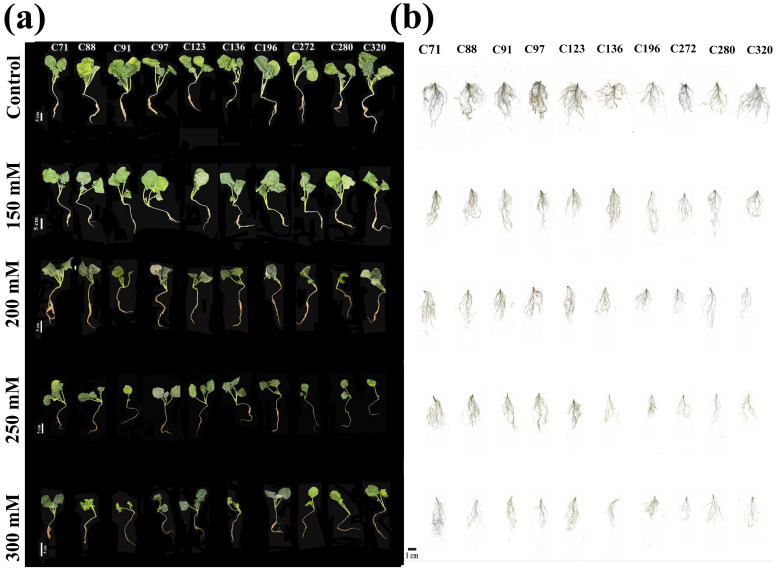
Salt stress induced morphological variations in control, 150, 200, 250, and 300 mM of NaCl as a salinity solution in different rapeseed varieties. (**a**) Whole seedling phenotype and (**b**) root system architecture of all studied varieties.

**Figure 2:**
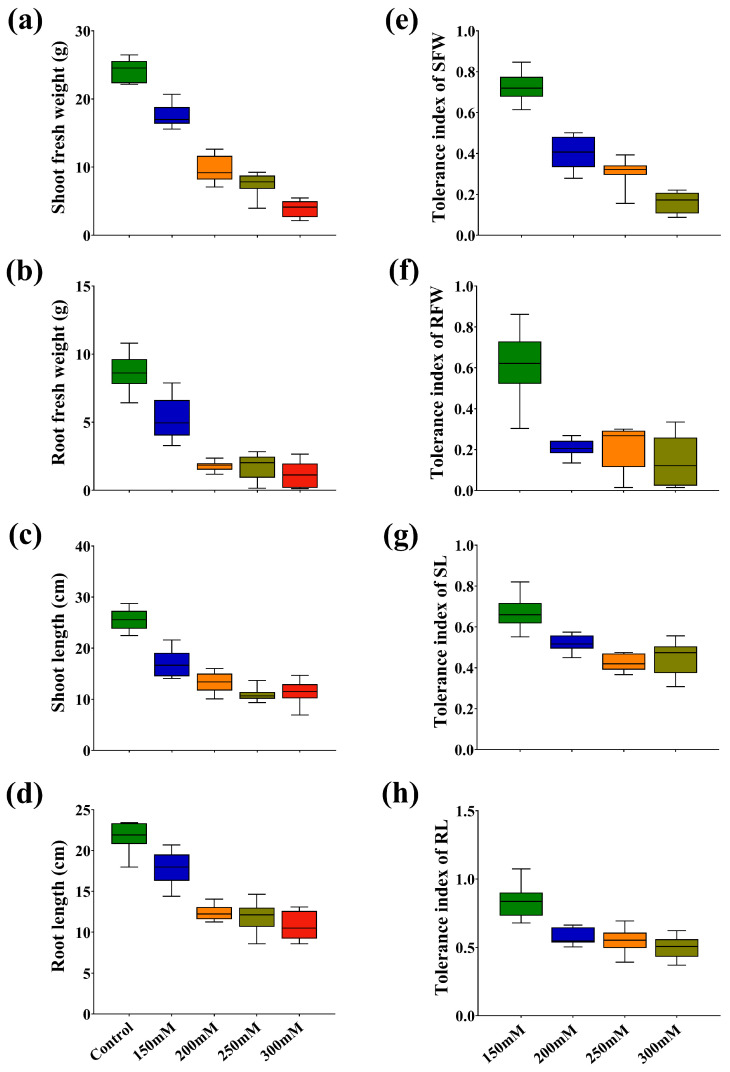
Box and whisker charts showing salt stress-induced variation of ten varieties in (**a**) shoot fresh weight, (**b**) root fresh weight, (**c**) shoot length, (**d**) root length, (**e**) tolerance index of shoot fresh weight, (**f**) tolerance index of root fresh weight, (**g**) tolerance index of shoot length, and (**h**) tolerance index of root length in rapeseed seedlings.

Ten promising rapeseed varieties were treated under various concentrations of NaCl to evaluate the negative impact of salt stress, which significantly reduced the shoot and root growth of seedlings with increasing concentrations, which was prominent in sensitive versus tolerant seedlings (Figure 1). Moreover, shoot fresh weight (SFW), root fresh weight (RFW), shoot dry weight (SDW), shoot length (SL), and root length (RL) were negatively influenced in studied varieties with increasing salt concentrations, which were highest decrement by 83.76, 86.64, 14.17, 54.94, and 50.22% at 300 mM versus control, respectively. Our results showed that C272 recorded the highest decrement in SFW by 84.36 and 88.96%, while C71 noted the lower reduction by 66.60 and 79.10% at 250 and 300 mM versus control, respectively. Moreover, RFW was reduced in C272, C71, and C97 by 98.04, 70.55, and 71.99% at 250 mM, while reduced by 98.59 and 74.87% in C272 and C71 at 300 mM versus the control, respectively (Figure 2a–h and Table S1).

**Figure 3:**
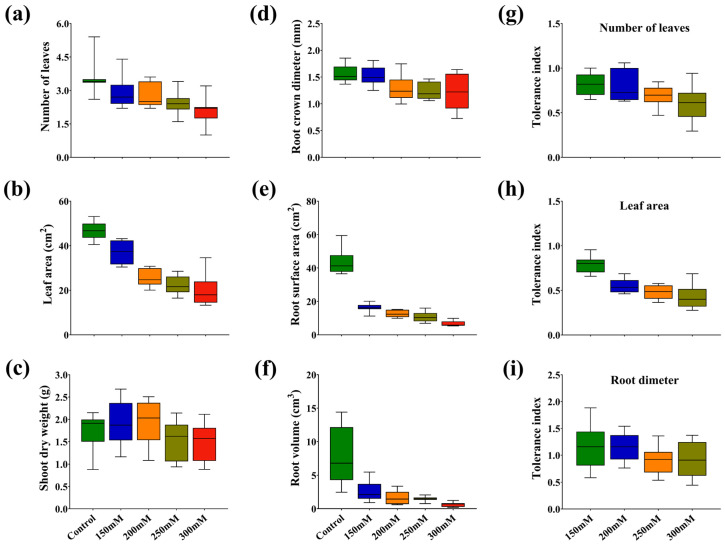
Box and whisker charts showing salt stress-induced variation of ten varieties in (**a**) number of leaf, (**b**) leaf area, (**c**) relative water content, (**d**) root crown diameter, (**e**) root surface area, (**f**) root volume, (**g**) tolerance index of number of leaves, (**h**) tolerance index of leaf area and (**i**) tolerance index of root dimeter in rapeseed seedlings.

At 250 mM, SL was reduced in C272, C280, and C71 by 37.32, 37.47, and 34.30% versus control, respectively. Moreover, RL noted the highest reduction in C272, C280, and C71 by 61.64, 60.01, and 40.46% at 300 mM versus control, respectively. Moreover, SDW, root surface area, root volume, leaf area, number of leaves, and root crown diameter were significantly decreased with increasing salt concentration, especially at 300 mM by 14.17, 84.29, 88.62, 57.08, 41.24, and 22.74%, respectively, versus control. Similarly, the number of leaves, leaf area, root crown diameter, and SDW were significantly reduced by 59.25, 53.71, 19.95, and 45.53% (C272); 29.41, 31.29, 8.426, and 6.318% (C71) at 300 mM versus control, respectively (Figure 3, Tables S1 and S2).

### 3.2. Variation in Photosynthetic Pigments, Amino Acids, and Osmolytes in Rapeseed Seedlings Under Stress Conditions

**Figure 4:**
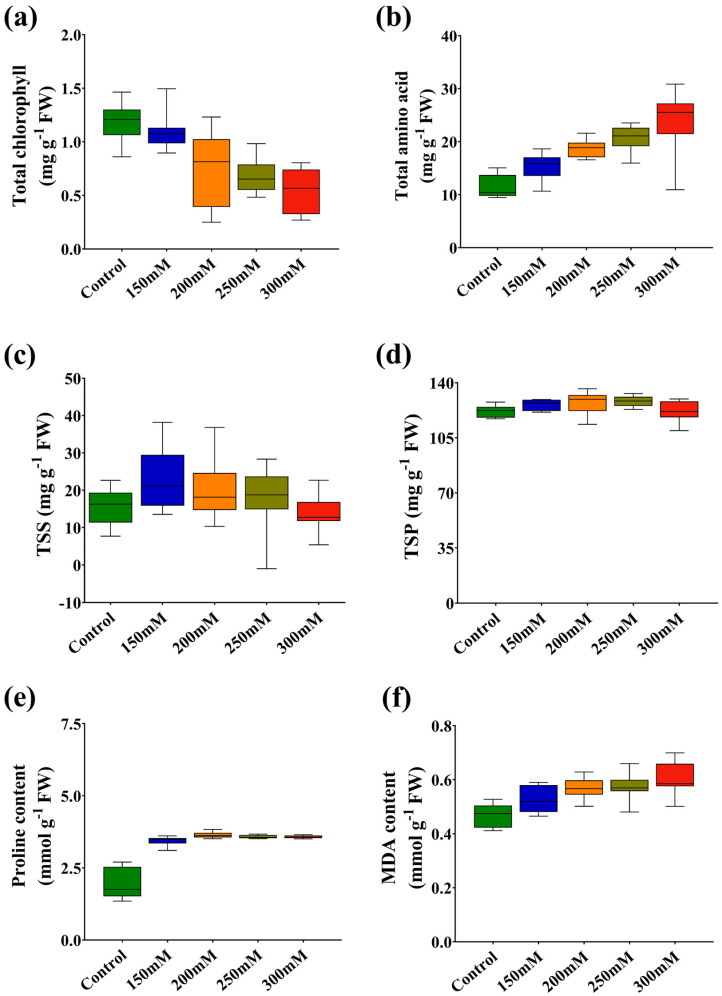
Box and whisker charts showing salt stress-induced variation of ten varieties in (**a**) total chlorophyll, (**b**) total amino acids, (**c**) total soluble sugar, (**d**) total soluble protein, (**e**) proline, and (**f**) MDA contents in rapeseed seedlings.

Total chlorophyll content was decreased in salt-treated seedlings with increasing salt concentration, where it was recorded as 77.92 and 42.28% in C272 and C71 at 300 mM versus the control, respectively. Moreover, amino acid accumulation is a vital plant stress response to degenerate ROS that reduces oxidative stress, where it was significantly increased with increasing salt concentration, especially at 300 mM by 54.53 and 221.91% in C272 and C71, respectively, versus the control (Figure 4a,b, and Table S3). Total soluble sugars (TSS) content was higher in C97 and C320 by 44.64 and 74.64%; meanwhile, total soluble proteins (TSP) content was higher in C71 and C123 by 15.16 and 12.86% at 300 mM versus control, respectively (Figure 4c,d and Table S3). Conversely, proline content was increased at all stress concentrations, with the highest increment in C272, C320, and C71 by 24.53, 167.5, and 157.6% at 300 mM versus control, respectively. The current results showed that MDA content was significantly increased with increasing stress concentration, especially at 300 mM by 43.58 and 39.18% in C272 and C88, respectively. Meanwhile, it was recorded as a lower increment by 9.141 and 9.302% in C71 and C196 under 300 mM versus control, respectively (Figure 4e,f and Table S3).

### 3.3. Time-Specific Growth Responses of Tolerant and Sensitive Varieties Under Salt Stress Conditions

**Figure 5:**
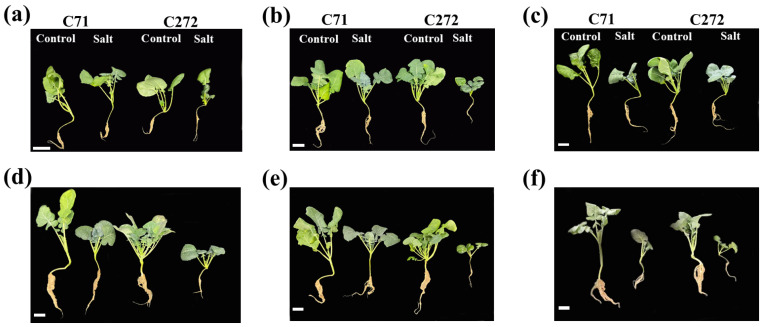
Salt stress induced morphological variations in rapeseed seedlings at different time points (**a**) 1st week, (**b**) 2nd week, (**c**) 3rd week, (**d**) 4th week, (**e**) 5th week, and (**f**) 6th week.

Based on morpho-physiochemical traits analysis of ten rapeseed varieties, C71 and C272 were selected as tolerant and sensitive varieties to study stress responses during six weeks (weekly time points) in the leaf, petiole, stem, and root of seedlings under 250 mM of NaCl. Our results displayed a substantial reduction in rapeseed seedlings’ growth in terms of shoot and root upon 250 mM, which was more prominent in sensitive seedlings at different time points (Figure 5). Moreover, SFW decreased with increasing growing time under stress conditions from the 1st week (29.63 and 35.01%) to the 6th week (68.72 and 89.16%) in C71 and C272 versus control, respectively. Moreover, SL, RD, and RFW decreased by 47.71, 95.71, and 67.43% (C71), and 56.31, 77.73, and 98.03% (C272) at the 6th week versus control, respectively. Similarly, RL and the number of leaves were decreased by 2.881 and 23.52% (C71), 6.672 and 29.16% (C272) at the 1st week, and 54.65 and 28.07% (C71), 68.46 and 42.11% (C272) at the 6th week, respectively, versus control (Table 1).

### 3.4. Temporal Ion Dynamics of Na^+^ and K^+^ Contents in Rapeseed Seedlings Under Salt Stress Conditions

**Figure 6:**
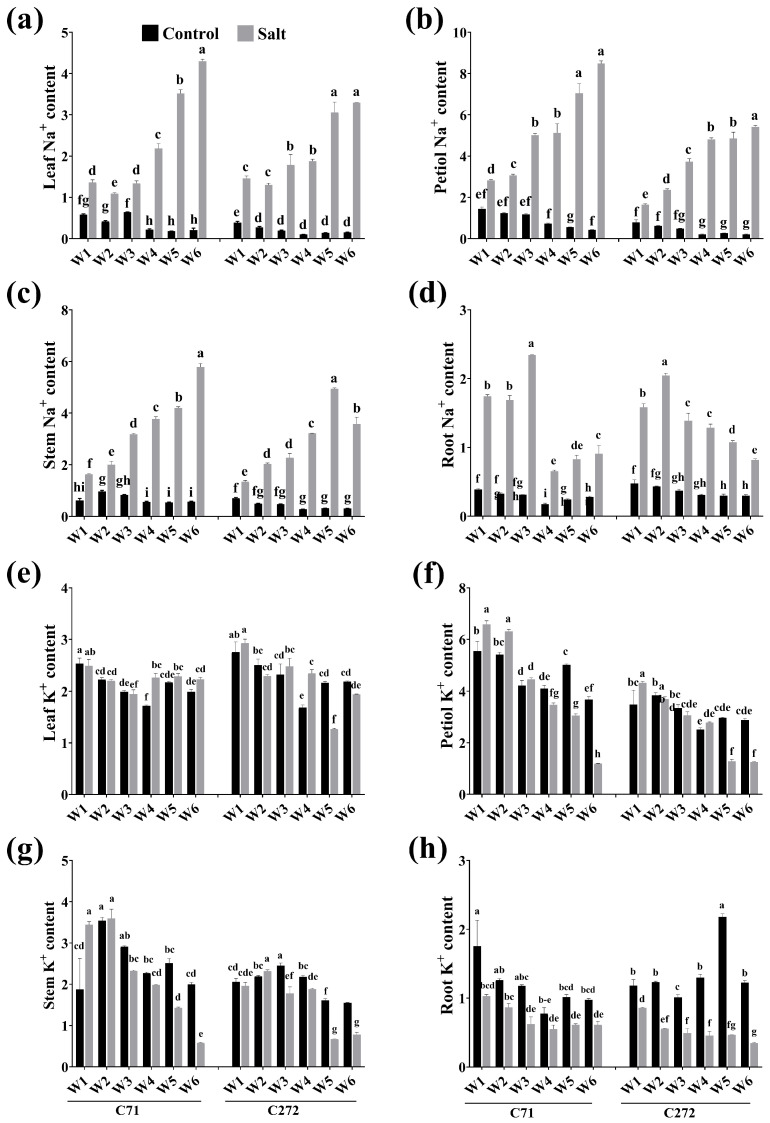
Effect of salt stress on (**a**–**d**) sodium ion (Na^+^) and (**e**–**h**) potassium ion (K^+^) contents in leaf, petiole, stem, and root of rapeseed seedling during the early seedling stage. Bars represent ± SE of three replicates. The different letters indicate significant differences at *p* < 0.05 using Duncan’s multiple range tests. W1 to W6 represents 1st week to the 6th week of growing time after salt treatment.

The Na^+^ content was increased with the exposure time compared to the control, which was reduced in the 2nd week compared to the 1st week by 19.10 and 10.01% in C71 and C272 seedling leaves, then it increased afterward. Moreover, at the 6th week, Na^+^ content was highest in leaves, petioles, and stems by 21.21, 20.01, and 37.11% in C71 and 7.110 and 11.02% in C272, respectively; however, it decreased by 27.01% in the stems compared to the 5th week. Meanwhile, Na^+^ was accumulated higher in the roots by 34.31% (C71) in the 3rd week and 29.31% (C272) in the 2nd week compared to accumulation in the 1st week. It was decreased by 72.11% (C71) in the 4th week compared to the 3rd week, while it decreased by 32.52% (C272) in the 3rd week versus the 2nd week. Additionally, in seedling leaves, Na^+^ was higher by 232.7% (C71) at the 4th week and 78.08% (C272) in the 3rd week, while it increased by 114.05 and 168.4% (petiole) and 218.6 and 127.08% (stem) in C71 and C272 in the 3rd week versus seedlings’ roots, respectively (Figure 6a–d).

In leaves, K^+^ accumulation was increased in C71 by 15.98% and decreased in C272 by 5.421% at the 4th week versus the 3rd week. Moreover, it was reduced by 4.188 and 14.77% in the petiole while increasing by 4.401 and 18.82% in the stem in the 2nd week versus the 1st week in C71 and C272, respectively. In roots, the accumulation was significantly increased at the 4th week by 21.81 and 15.66% versus the 3rd week in C71 and C272, respectively. On the other side, the K^+^ content was increased in leaves by 173.1 and 226.2% at the 3rd week in C71 and C272, respectively, versus roots. It was highest in the 2nd week in the stem by 335.3% (C71) and 207.7% (C272). While it was highest by 642.5, 616.1, and 435.7% (C71) and 413.2, 353.1, and 289.1% (C272) at 1st week, 2nd week, and 3rd week in the petiole versus roots, respectively (Figure 6e–h).

### 3.5. Temporal Overview of Osmolyte Variation in Rapeseed Seedlings Under Salt Stress Conditions

**Figure 7:**
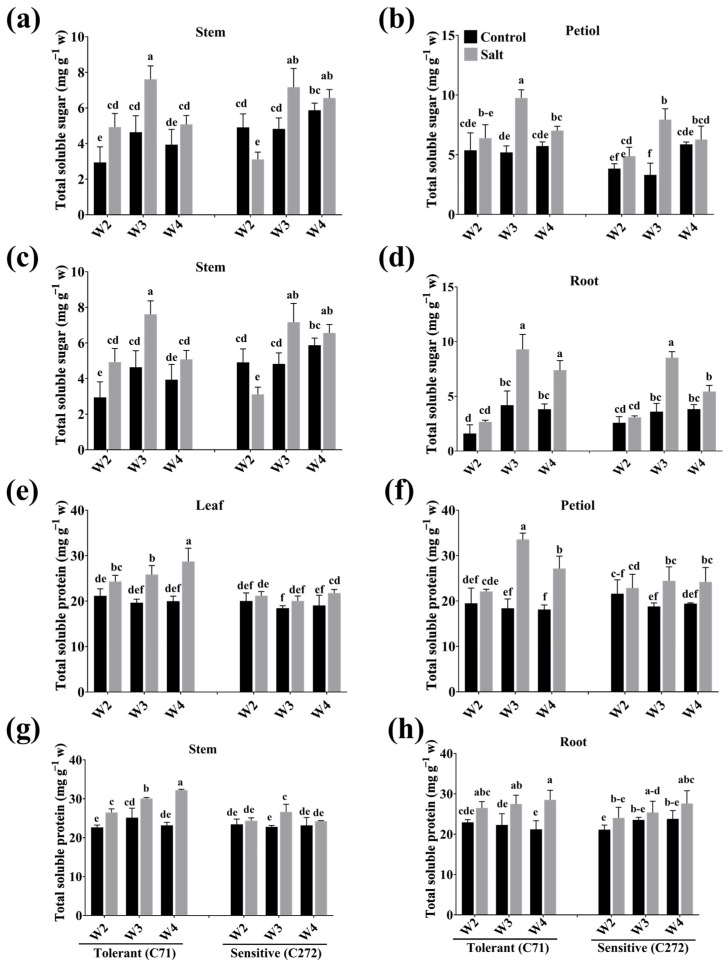
Effect of salt stress on (**a**–**d**) total soluble sugar (TSS) and (**e**–**h**) total soluble protein (TSP) contents in leaf, petiole, stem, and root of rapeseed seedling during the early seedling stage. Bars represent ± SE of three replicates. The different letters indicate significant differences at *p* < 0.05 using Duncan’s multiple range tests. W2 to W4 represents the 2nd week to the 4th week of growing time after salt treatment.

Herein, TSS content was increased under stress conditions, which was 65.11, 44.87, and 45.66% (leaves) and 40.14, 51.96, and 48.36% (roots) in C71, while 40.32, 38.61, and 16.31% (leaves) and 15.99, 59.49, and 29.59% (roots) in C272 at the 2nd, 3rd, and 4th weeks versus control, respectively. While it was highest at 3rd week by 46.81 and 58.39% (petiole) and 40.13 and 32.71% (stem) in C71 and C272 versus control, respectively (Figure 7a–d). Meanwhile, TSP content was increased in leaves by 12.95 and 5.436% (2nd week), 24.00 and 8.032% (3rd week), and 30.43 and 12.65% (4th week) in C71 and C272, respectively, versus control. It was highest in the 3rd week by 45.23 and 23.16% in the petiole of C71 and C272 under stress conditions versus control. Whereas the highest increment was 28.10% at the 4th week of C71 and 14.57% at the 3rd week in the stem of C272 under salt stress versus control. Moreover, TSP was elevated by 13.51, 18.85, and 25.60% (C71), and 12.11, 7.241, and 11.76% (C272) in the roots at the 2nd, 3rd^,^ and 4th weeks of salinity treatment versus control, respectively (Figure 7e–h).

### 3.6. Temporal Overview of Proline and MDA in Rapeseed Seedlings Under Salt Stress Conditions

**Figure 8:**
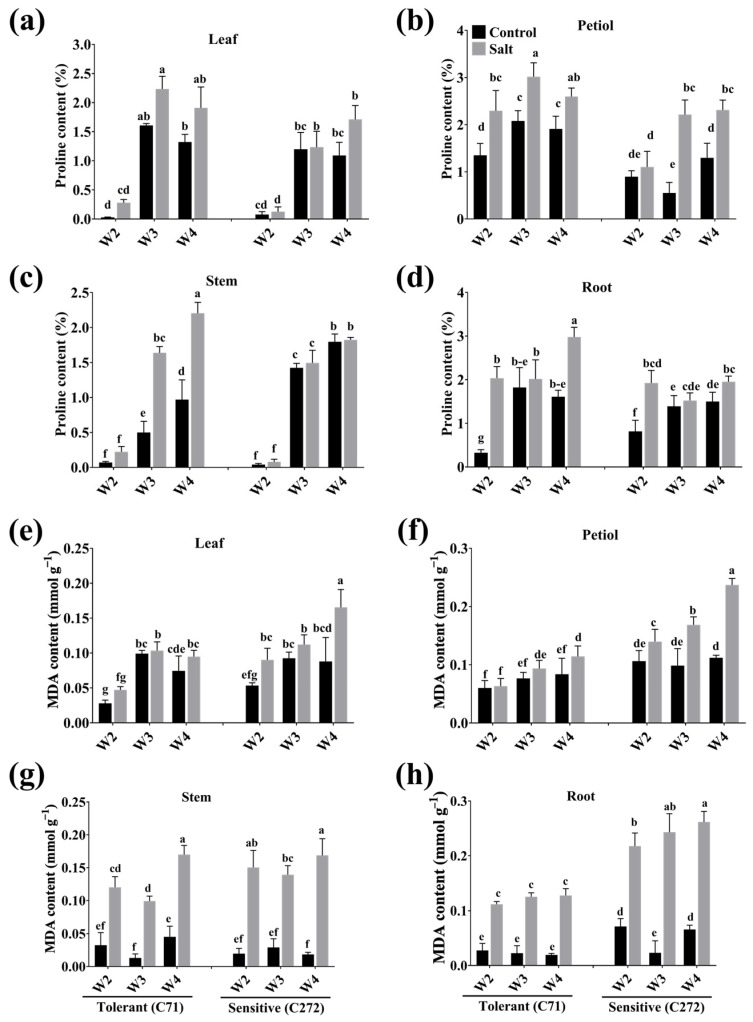
Effect of salt stress on (**a**–**d**) proline and (**e**–**h**) malonaldehyde (MDA) contents in leaf, petiole, stem, and root of rapeseed seedling during the early seedling stage. Bars represent ± SE of three replicates. The different letters indicate significant differences at *p* < 0.05 using Duncan’s multiple range tests. W2 to W4 represents the 2nd week to the 4th week of growing time after salt treatment.

In leaves and roots, proline content was increased under stress conditions, especially at the 3rd week, by 84.01 and 57.61% (leaves) and 10.39 and 36.84% (roots) in C71 and C272 versus control, respectively. Moreover, it was highest by 41.16% (2nd week) in C71 and 75.07% (3rd week) in C272 in petiole and 69.81% (3rd week) in C71 and 48.87% (2nd week) in C272 in stem versus control, respectively (Figure 8a–d). On the other hand, MDA content was increased in leaves by 40.76, 4.361, and 21.05% of C71 and 40.90, 17.49, and 46.85% of C272 at the 2nd, 3rd, and 4th weeks of salinity treatment, respectively, versus control. Besides, it was significantly increased in petiole and stem, especially at the 3rd week, by 5.467 and 86.89% in C71, while 41.52 and 79.09% in C272, respectively, versus the control under salt stress conditions. In the roots, MDA was increased under stress conditions by 82.05 and 90.42% at the 3rd week of salinity treatment versus control in C71 and C272, respectively (Figure 8e–h).

## 4. Discussion

Salt stress is one of the crucial abiotic stresses that significantly affects agricultural productivity; therefore, a deep understanding of differential stress response provides a basic foundation for rapeseed breeders with improved salt tolerance and adaptability [10]. The current study aimed to evaluate the effect of varying levels of salt stress on rapeseed seedlings by comprehending the morpho-physiochemical stress responses. Seedling establishment is one of the sensitive stages under salt stress, significantly affecting plant growth and development [24]. Moreover, reduced growth is a primary symptom of salt stress, highlighting the importance of cultivar selection for tolerance in saline environments [8]. Salt stress decreased the shoot length and leaf area due to higher osmotic and ionic imbalance, which reduced cell division and elongation [45]. Our results clearly showed that salt stress intensity reduced the seedling growth, especially with increasing salt concentration, as evidenced by the lower mean value of growth-related traits compared to the control. Besides, the progressive reduction of growth indicated the dose-dependent nature of salt stress, where higher stress led to increasing damage to plants, indicating the diversity of salt tolerance that existed among various rapeseed cultivars [16]. Salt stress reduced root growth by affecting osmotic and ionic imbalance, generating various oxidants and free radicals that disrupt the membrane potential, integrity, and permeability [8]. Besides, it disrupts photosynthesis and protein synthesis in *Brassica* seedlings [46].

Photosynthetic pigments are essential components in photosynthetic machinery; moreover, chlorophyll is an important photosynthetic pigment that exists in different forms and abundances [47]. It facilitates efficient light harvesting at different wavelengths, allowing photosynthetic organisms to adapt to different environments [48]. Moreover, salt stress significantly reduces the photosynthetic process by affecting the electron transport chain and oxidative damage that disrupts cellular integrity and chlorophyll structure, thus disrupting metabolic processes in plants [10]. Our investigation revealed that photosynthetic pigments of rapeseed seedlings were negatively affected under salt stress, which was to a lesser extent in tolerant than sensitive seedlings, and increased with higher stress levels. It indicates that tolerant seedlings might exhibit higher photorespiratory activity, nitrate assimilation, and higher cyclic electron flow [49].

Osmotic adjustment is one of the essential strategies for stress tolerance, where organic solutes, including TSS and TSP, reduce the osmotic damage under unfavorable conditions in plants [50,51]. Plants synthesize and accumulate soluble sugars in the cytosol that exhibit a role as nutrients, organic solutes, and metabolic signaling molecules in different environmental adversities [32]. It works as an important osmotic substance under salt exposure, which regulates osmotic balance and turgor of cells for proper development [52]. It is also involved in the regulation of growth and stress-related genes by hexokinase-dependent and/or hexokinase-independent pathways [32]. Soluble proteins are involved in osmoregulation that protects cellular membranes, structural proteins, and enzymes from excessive Na^+^ accumulation [53]. Besides, it also works as an enzyme complex to regulate the synthesis of soluble sugar for osmoregulation [54]. Salt-tolerant plants maintain basic metabolic activity and induce stress-responsive proteins such as dehydrins and late embryogenesis abundant proteins to protect cellular structure from Na^+^ toxicity and Na^+^-induced dehydration [55]. Moreover, plants accumulate organic solutes to cope with salt-induced osmotic stress, which retain cellular water and stabilize membranes and enzymes [53]. Our results showed that TSS and TSP contents increased in all studied rapeseed seedlings under various salinity concentrations. Moreover, they increased the accumulation with increasing salt concentration, where it accumulated higher in tolerant versus sensitive seedlings. On the other side, TSS and TSP contents were higher at the 3rd week in tolerant seedling shoots, indicating a plant adaptive response to scavenge ROS generation [56].

Amino acids as secondary metabolites play a vital role in various downstream metabolic processes in tolerant plants under salinity conditions [57]. Previous studies indicate that the rearrangement of nitrogen metabolism under salt stress could accumulate more amino acids as the compatible solute rather than the components of proteins [58]. Our results showed that total amino acid content was enhanced in tolerant seedlings, which might modulate plant metabolic mechanisms and cellular homeostasis to improve the antioxidant system and reduce toxic ion accumulation [37]. Moreover, amino acids work as precursors of the synthesis of various signaling molecules, and their accumulation in the cytoplasm improves cell water potential, which reduces osmotic damage and improves stress tolerance [57,59]. Proline is a vital osmo-protectant and signaling molecule that protects cellular membranes, proteinaceous enzymes, and protein configurations from damage to improve stress tolerance [35]. In tolerant plants, osmotic stress induced by high salinity triggers proline accumulation as an osmoprotectant to maintain cellular water balance by lowering osmotic potential [40]. Besides, proline stabilizes proteins and membranes, protects cellular structures from denaturation, and acts as a powerful antioxidant that scavenges ROS generated to preserve metabolic activity [60]. It was enhanced in rapeseed seedlings, especially in tolerant seedlings at the 3rd week, which reduces oxidative damage and maintains metabolic processes under salt stress conditions [61].

In plants, the membrane system is the initial site for sensing stress-induced cellular damage, manifested as plant lipid peroxidation, where MDA is an essential physiological index of plant damage extent under stress conditions [62]. Previous reports showed that MDA influences the structure of the thylakoid membrane and leads to chlorophyll degradation, thus affecting the photosynthetic apparatus in plants [63]. Oxidative stress is a toxic effect of free radicals, including H_2_O_2_ and O_2_^•–^, that leads to lipid peroxidation in the cellular membrane and its permeability, which alters the electron transport chain through protein degradation and affects the photosystem [56,64]. Our results showed that MDA content was enhanced under stress conditions in rapeseed seedlings, which was highly increased in sensitive as compared to tolerant seedlings. In salt-tolerant plants, MDA levels are lower than in sensitive plants, which might be due to stronger antioxidant defenses and higher concentrations of protective solutes [65]. Additionally, by decreasing MDA accumulation, salt-tolerant plants maintain better membrane integrity, reduce oxidative injury, and sustain essential physiological processes, especially with water deficit induced by salt stress [51]. To encounter oxidative injury, plants exhibit comprehensive antioxidative systems, including osmotic substances that scavenge ROS without the conversion to any destructive effect, thereby reducing MDA level [7].

The intracellular ionic (Na^+^/K^+^) homeostasis is essential for appropriate physiochemical mechanisms for plant growth, especially under salt stress [66]. Plants maintain a complex ion flux to prevent ionic toxicity through various mechanisms for ionic balance, especially ion transportation via proton pumps, ion transporters, and channels that stabilize the K^+^/Na^+^ ratio in the cytosol [67]. Excessive Na^+^ causes ionic toxicity, which leads to nutritional imbalance through competing with beneficial ions, thus altering the cellular biochemical process and reducing plant biomass [31]. Under salt stress, ionic toxicity may be postponed or avoided via an adaptive mechanism of Na^+^ exclusion for plant survival through balancing K^+^ uptake [51]. Additionally, tolerant seedlings with higher Na^+^ accumulation exhibited coordinated regulation of Na^+^ sequestration, controlled Na^+^ translocation, and sustained K^+^ retention besides higher osmotic adjustment [68,69]. Our study showed higher K^+^ accumulation in all parts of seedlings in C71 than in C272, reducing Na^+^ toxicity for stress tolerance. In addition, K^+^ is a vital macronutrient required in large amounts from the soil and transferred to the plant, improving growth and development [70]. K^+^ is involved in several cellular physiological and molecular mechanisms; however, excessive Na^+^ content reduced K^+^ uptake, leading to a deficient K^+^ level that restricted cellular development under higher salinity [71,72].

**Figure 9:**
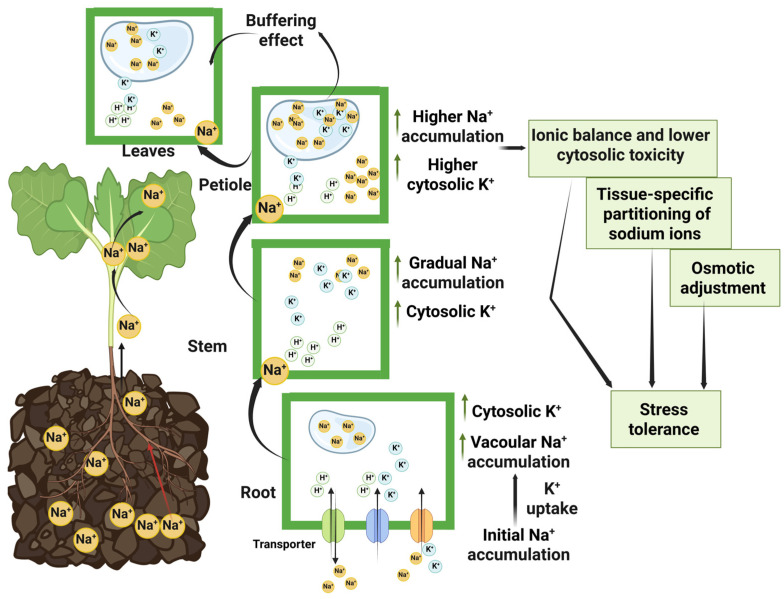
Schematic diagram indicating ionic movement in different parts of rapeseed seedlings under salt stress conditions. Green arrows indicate an increase, while black arrows represent general directional indication.

The content of K^+^ was increased in the seedlings above-ground parts, especially the petiole in the C71 compared to the C272, indicating the role of K^+^ in maintaining the ion ratio and reducing the effect of toxic ions for stress tolerance [7]. Our study showed higher K^+^ content in root-tolerant seedlings, resulting in K^+^ homeostasis, which is essential for enzyme activity, ionic homeostasis, and pH balance [73]. Our results showed that Na^+^ initially accumulates in roots and enhances K^+^ levels in tolerant seedlings, which leads to ion homeostasis, which might be due to Na^+^ substitution for K^+^ in the vacuole, which increases the cytosolic K^+^ availability, thus improving stress tolerance [74,75]. Moreover, higher Na^+^ accumulation in C71 leads to stress tolerance, associated with controlled Na^+^ movement from roots to petioles along with managed K^+^ levels, which help buffer the leaves, leading to ensuring stability and maintaining cellular functions [74]. Besides, gradual Na^+^ movement from roots to stems ensures even distribution, preventing localized toxicity through higher K^+^ levels in stems, which maintain cytosolic ion homeostasis and support structural integrity through efficient ion transport using ion transporters and ion pumps [65], as shown in Figure 9.

## 5. Conclusions

Salt stress intensity reduced the seedling growth, as evidenced by the lower mean value of growth traits; besides, the progressive reduction of growth indicated the dose-dependent nature of salt stress. Various rapeseed varieties showed varied tolerance levels with increasing salt stress conditions. Moreover, osmolyte accumulation, including TSS, TSP, and proline contents, was enhanced, with decreasing MDA levels, especially in tolerant seedlings. Based on the initial screening, C71 (tolerant) and C272 (sensitive) were selected for detailed physiological and ionic analysis. The tolerant seedling of C71 showed higher osmolyte contents (TSS, TSP, and proline, especially at the 3rd week in different seedling parts), indicating higher osmotic adjustment as compared to sensitive seedlings. With increasing growing time, Na^+^ and K^+^ contents were differentially accumulated in different tissues under 250 mM NaCl. Moreover, Na^+^ initially accumulates in roots and enhances K^+^ levels in tolerant seedlings, besides K^+^ accumulating higher in the roots of tolerant seedlings. Additionally, Na^+^ accumulation was increased in different above-ground parts of seedlings, especially petioles, suggesting Na^+^ translocation from roots to above-ground parts. Conclusively, current findings provide a framework for fundamental time-course morpho-physiochemical analyses, which help identify salt-tolerant and salt-sensitive characteristics for developing a salt-tolerance breeding program for rapeseed cultivars.

## Figures and Tables

**Table 1:** Effect of salt stress on the rapeseed seedlings’ growth-related traits during different weeks of stress.

Treatments		Time (h)	SFW	SL	RD	RFW	RL	No of Leaf
Tolerant variety (C71)	Control	W1	6.562 ± 1.27 ^hij^	17.84 ± 0.35 ^d–h^	2.566 ± 0.25 ^efg^	0.842 ± 0.05 ^fgh^	12.25 ± 0.35 ^def^	4.533 ± 0.12 ^cde^
		W2	13.07 ± 0.44 ^e^	24.36 ± 1.59 ^b^	2.676 ± 0.21 ^def^	2.236 ± 0.35 ^defg^	18.22 ± 1.08 ^abc^	4.666 ± 0.31 ^cde^
		W3	17.41 ± 1.42 ^d^	28.6 ± 1.17 ^a^	3.694 ± 0.36 ^cde^	2.738 ± 0.57 ^de^	20.76 ± 2.07 ^ab^	4.533 ± 0.12 ^cde^
		W4	18.78 ± 1.01 ^cd^	30.9 ± 1.77 ^a^	4.515 ± 0.53 ^bc^	5.096 ± 0.80 ^c^	20.43 ± 1.10 ^ab^	4.412 ± 0.34 ^cde^
		W5	24.19 ± 2.26 ^ab^	30.4 ± 1.47 ^a^	6.18 ± 1.04 ^a^	9.101 ± 1.45 ^b^	22.73 ± 1.66 ^a^	4.833 ± 0.28 ^cde^
		W6	27.21 ± 0.83 ^a^	30.1 ± 0.55 ^a^	5.333 ± 0.72 ^ab^	11.05 ± 1.21 ^a^	22.96 ± 2.24 ^a^	3.801 ± 0.34 ^de^
	Salt stress	W1	4.617 ± 0.13 ^hij^	17.97 ± 1.51 ^d–h^	2.241 ± 0.13 ^fgh^	0.540 ± 0.01 ^fgh^	11.91 ± 0.62 ^d–g^	3.466 ± 0.30 ^de^
		W2	7.884 ± 0.88 ^hi^	19.2 ± 1.01 ^c–f^	1.873 ± 0.07 ^fgh^	0.643 ± 0.08 ^fgh^	16.06 ± 2.02 ^bcd^	3.211 ± 0.05 ^de^
		W3	12.46 ± 1.05 ^ef^	14.63 ± 1.76 ^g–j^	1.965 ± 0.17 ^fgh^	1.363 ± 0.11 ^d–h^	15.23 ± 1.02 ^cde^	3.533 ± 0.11 ^de^
		W4	7.656 ± 0.98 ^hi^	16.36 ± 1.01 ^f–i^	2.16 ± 0.31 ^fgh^	1.01 ± 0.13 ^e–h^	13.12 ± 1.14 ^def^	3.866 ± 0.50 ^de^
		W5	8.94 ± 1.03 ^e–h^	17.53 ± 1.37 ^e–h^	2.496 ± 0.18 ^e–h^	1.26 ± 0.09 ^d–h^	13.16 ± 1.04 ^def^	3.466 ± 0.30 ^de^
		W6	8.513 ± 0.922 ^fgh^	15.78 ± 0.99 ^fg^	1.736 ± 0.26 ^fgh^	0.473 ± 0.07 ^gh^	10.41 ± 1.10 ^e–h^	2.733 ± 0.71 ^e^
Sensitive varierty (C272)	Control	W1	8.092 ± 0.72 ^gh^	18.47 ± 0.92 ^d–g^	1.899 ± 0.28 ^fgh^	0.803 ± 0.09 ^fgh^	14.74 ± 1.24 ^cde^	4.8 ± 0.87 ^cde^
		W2	12.393 ± 1.69 ^efg^	21.73 ± 1.41 ^bcd^	2.673 ± 0.52 ^def^	2.280 ± 0.56 ^def^	20.26 ± 0.64 ^ab^	7.212 ± 1.05 ^b^
		W3	19.72 ± 0.72 ^cd^	21.1 ± 0.79 ^b–e^	3.838 ± 0.21 ^cd^	2.86 ± 0.44 ^d^	21.73 ± 3.82 ^a^	7.601 ± 0.6 ^ab^
		W4	22.80 ± 2.81 ^bc^	21.5 ± 1.32 ^b–e^	3.838 ± 0.21 ^cd^	6.121 ± 0.92 ^c^	20.45 ± 2.86 ^ab^	9.812 ± 1.21 ^a^
		W5	24.07 ± 2.69 ^ab^	22.93 ± 2.50 ^bc^	5.706 ± 0.39 ^ab^	5.36 ± 0.97 ^c^	20.86 ± 2.02 ^ab^	9.866 ± 1.85 ^a^
		W6	27.65 ± 2.01 ^a^	20.06 ± 1.52 ^c–f^	5.78 ± 0.73 ^a^	8.98 ± 0.66 ^b^	21.66 ± 1.25 ^a^	6.666 ± 1.52 ^bc^
	Salt stress	W1	5.26 ± 0.211 ^hij^	13.42 ± 0.77 ^ij^	1.502 ± 0.13 ^fgh^	0.59 ± 0.03 ^fgh^	13.75 ± 0.29 ^c–f^	3.4 ± 0.01 ^de^
		W2	4.656 ± 0.67 ^hij^	14.13 ± 1.02 ^hij^	1.509 ± 0.07 ^fgh^	0.488 ± 0.02 ^gh^	12.26 ± 1.21 ^def^	4.733 ± 0.81 ^cde^
		W3	6.399 ± 1.94 ^hij^	12.96 ± 0.96 ^ij^	1.536 ± 0.21 ^fgh^	1.02 ± 0.21 ^e–h^	14.4 ± 0.98 ^c–f^	5.333 ± 0.64 ^bcd^
		W4	5.74 ± 1.49 ^hij^	14 ± 0.5 ^hij^	1.48 ± 0.15 ^fgh^	0.201 ± 0.06 ^h^	9.712 ± 0.75 ^fgh^	4.466 ± 0.23 ^cde^
		W5	3.713 ± 0.433 ^ij^	10.56 ± 1.26 ^jk^	1.323 ± 0.25 ^gh^	0.190 ± 0.04 ^h^	7.266 ± 0.68 ^gh^	3.466 ± 0.31 ^de^
		W6	2.996 ± 0.26 ^j^	8.766 ± 0.80 ^k^	1.286 ± 0.23 ^h^	0.176 ± 0.06 ^h^	6.833 ± 0.94 ^h^	3.866 ± 0.71 ^de^
ANOVA								
Time (T)			98.33 **	16.46 **	41.48 **	106.9 **	12.64 **	9.321 **
Variety (V)			12.60 **	266.8 **	20.39 **	20.99 **	9.081 *	138.9 **
Salt stress (S)			1353 **	1009 **	605.2 **	969.8 **	441.5 **	175.5 **
TxV			4.841 *	6.632 **	2.361 ^ns^	9.512 **	5.421 **	5.782 **
TxS			83.39 **	36.04 **	44.37 **	122.7 **	27.13 **	5.413 **
VxS			54.84 **	11.92 *	5.401 *	1.641 ^ns^	16.62 **	47.73 **
TxVxS			2.842 *	12.28 **	0.601 ^ns^	6.280 **	1.042 ^ns^	5.894 **

The data presented are the mean values of three replicates. The difference in letters indicates significant differences at (*p* < 0.05) using Duncan’s multiple range tests. SFW: shoot fresh weight, SL: shoot length, RD: root crown diameter, RFW: root fresh weight, RL: root length, and No. of leaf: number of leaves. W1 to W6 represents 1st week to the 6th week of growing time after salt treatment. Asterisks represent * *p* < 0.01, ** *p* < 0.001, and ^ns^ non-significant.

## Data Availability

The original contributions presented in this study are included in the article. Further inquiries can be directed to the corresponding author.

## Supplementary Materials

The following supporting information can be downloaded at: https://www.mdpi.com/article/10.3390/plants15040661/s1, Table S1: Effect of salt stress on growth traits of rapeseed seedlings in different varieties.; Table S2: Effect of salt stress on No of leaves, leaf area, root surface area, and root volume of rapeseed seedlings in different varieties; Table S3: Effect of salt stress on physiological traits of rapeseed seedlings in different varieties.
