# Supplementary material for: Comprehensive Morpho-Physiological Responses Underlying Salt Tolerance at Different Time Points in Brassica napus Seedlings
Source: Plants (Basel). 2026 Feb 22;15(4):661. doi: 10.3390/plants15040661 (PMC12944348; doi:10.3390/plants15040661)
Supplement: Supplementary file 1 [file plants-15-00661-s001.zip › plants-4112204-supplementary.pdf]

Table S1. Effect of salt stress on growth traits of rapeseed seedlings in different varieties.

Variety	SFW					RFW					SL				
	**Control**	**150 mM**	**200 mM**	**250 mM**	**300 mM**	**Control**	**150 mM**	**200 mM**	**250 mM**	**300 mM**	**Control**	**150 mM**	**200 mM**	**250 mM**	**300 mM**
**C280**	22.32±2.80^{cd}	18.74±2.46^{bc}	8.427±1.10^{e}	5.766±4.85^{d}	2.153±1.56^{f}	10.80±0.44^{a}	3.281±0.33^{f}	1.743±0.34^{b}	0.153±0.43^{f}	0.161±0.26^{d}	22.55±1.08^{e}	14.10±1.12^{f}	11.17±1.80^{ef}	9.96±1.17^{cd}	10.21±2.39^{e}
**C272**	25.35±3.65^{ab}	20.68±4.13^{a}	7.067±0.90^{f}	3.963±1.27^{e}	2.796±2.40^{ef}	8.517±0.27^{cd}	4.793±0.14^{cde}	1.753±0.24^{b}	0.166±0.44^{f}	0.121±0.31^{d}	22.48±0.82^{e}	14.09±0.63^{f}	10.09±2.01^{f}	9.343±0.13^{e}	6.927±0.10^{f}
**C136**	25.21±2.67^{ab}	16.44±0.72^{de}	12.62±0.86^{a}	9.231±6.64^{a}	4.391±0.85^{bc}	7.307±0.47^{e}	5.112±0.22^{cd}	1.946±0.24^{ab}	1.181±1.33^{e}	0.192±0.14^{d}	24.22±0.83^{d}	16.02±0.41^{e}	11.88±1.24^{de}	11.37±1.36^{bc}	12.05±1.87^{c}
**C196**	22.31±4.11^{cd}	17.50±1.60^{bcd}	10.86±2.33^{c}	8.923±1.78^{ab}	4.936±0.44^{ab}	8.803±0.23^{c}	4.181±0.07^{ef}	2.361±0.40^{a}	1.296±1.03^{e}	0.283±0.06^{cd}	26.80±0.98^{bc}	19.64±0.25^{b}	13.72±1.52^{bc}	10.49±0.13^{b-e}	10.95±2.07^{d}
**C320**	25.24±3.71^{ab}	18.99±1.82^{b}	8.721±1.90^{de}	7.786±4.24^{bc}	2.203±0.55^{f}	6.431±0.04^{f}	3.541±0.06^{f}	1.223±0.37^{c}	1.876±1.57^{d}	1.821±0.07^{b}	27.27±1.44^{bc}	18.11±1.07^{cd}	13.86±0.54^{bc}	11.53±0.66^{b}	13.58±2.09^{b}
**C88**	26.47±9.31^{a}	16.79±2.40^{de}	8.332±2.29^{e}	8.137±2.45^{abc}	5.171±0.35^{ab}	8.707±0.49^{cd}	4.676±0.03^{de}	1.176±0.56^{c}	2.363±1.18^{bc}	0.773±0.12^{c}	26.39±1.28^{c}	20.63±0.51^{a}	14.79±0.89^{ab}	9.667±0.60^{de}	14.67±0.47^{a}
**C123**	22.73±4.30^{cd}	16.66±8.07^{de}	7.731±6.03^{ef}	7.236±3.30^{c}	3.872±1.96^{cd}	8.253±1.32^{cd}	5.623±0.68^{c}	1.941±0.72^{ab}	2.173±1.03^{cd}	1.577±0.20^{b}	24.75±1.27^{d}	14.62±1.62^{f}	12.88±1.15^{cd}	10.21±0.94^{cde}	12.24±0.75^{c}
**C97**	23.83±3.09^{bc}	17.19±2.03^{cd}	11.42±1.59^{bc}	7.893±4.26^{bc}	4.946±0.57^{ab}	9.617±2.68^{b}	6.553±0.41^{b}	1.972±0.19^{ab}	2.693±1.13^{ab}	1.473±0.33^{b}	24.34±0.78^{d}	17.33±0.34^{d}	13.09±0.71^{cd}	11.31±0.71^{bc}	12.77±0.84^{bc}
**C71**	26.24±7.44^{a}	15.56±3.08^{e}	12.39±0.44^{ab}	8.763±0.92^{ab}	5.483±1.41^{a}	9.645±0.90^{b}	7.883±0.04^{a}	2.041±0.04^{ab}	2.841±0.21^{a}	2.423±0.32^{a}	28.77±0.89^{a}	18.90±1.04^{bc}	16.03±0.48^{a}	13.65±0.96^{a}	10.99±2.10^{d}
**C91**	22.16±1.85^{d}	16.08±2.12^{de}	9.661±1.30^{d}	7.153±1.26^{c}	3.323±0.41^{de}	7.971±0.79^{de}	6.863±0.20^{b}	1.611±0.06^{bc}	2.393±0.20^{abc}	2.667±0.68^{a}	27.51±1.31^{b}	15.16±1.44^{ef}	15.81±0.79^{a}	10.66±0.72^{bcd}	10.55±1.78^{de}
	**RL**					**SDW**					**Root crown diameter**				
**C280**	23.33±0.78^{a}	14.42±1.74^{h}	11.54±1.16^{d}	9.923±2.39^{e}	9.331±1.14^{cd}	1.72±0.18^{abc}	2.441±0.33^{ab}	1.671±0.52^{cd}	1.743±0.93^{bc}	1.783±0.93^{ab}	1.503±0.10^{bcd}	1.342±0.15^{a}	1.381±0.13^{ab}	1.213±0.14^{ab}	0.726±0.05^{c}
**C272**	23.37±0.54^{a}	16.35±2.26^{fg}	11.24±0.41^{d}	8.623±0.10^{f}	8.963±0.50^{cd}	1.97±0.23^{ab}	1.486±0.25^{ef}	1.511±0.54^{de}	1.061±0.15^{e}	1.073±0.39^{de}	1.453±0.14^{cd}	1.473±0.04^{a}	1.121±0.28^{b}	1.063±0.09^{a}	1.163±0.21^{abc}
**C136**	23.33±0.28^{a}	18.74±1.50^{cd}	13.03±1.25^{ab}	12.01±1.87^{cd}	9.483±1.38^{cd}	1.99±0.37^{ab}	1.161±0.07^{f}	1.543±0.32^{de}	1.221±0.53^{de}	0.883±0.07^{e}	1.366±0.06^{d}	1.251±0.03^{a}	1.163±0.04^{b}	1.106±0.28^{ab}	0.971±0.09^{bc}
**C196**	21.85±0.20^{b}	19.28±0.91^{bc}	11.70±1.77^{cd}	10.89±2.07^{de}	11.15±1.76^{b}	0.88±0.02^{d}	1.656±0.38^{def}	1.081±0.13^{e}	1.062±0.08^{e}	1.067±0.03^{de}	1.411±0.17^{d}	1.596±0.18^{a}	0.996±0.08^{b}	1.121±0.21^{ab}	1.041±0.10^{bc}
**C320**	21.15±1.15^{b}	20.68±1.18^{a}	12.78±0.32^{b}	12.53±2.09^{c}	12.48±1.89^{a}	1.27±0.39^{cd}	1.546±0.25^{ef}	1.961±0.13^{bcd}	0.941±0.97^{e}	1.746±0.19^{abc}	1.52±0.07^{bcd}	1.486±0.04^{a}	1.081±0.03^{b}	1.191±1.26^{ab}	0.751±0.09^{c}
**C88**	23.41±2.24^{a}	18.38±1.13^{cde}	11.59±0.87^{d}	14.64±0.477^{a}	9.807±0.48^{c}	2.15±1.36^{a}	1.786±0.52^{cde}	2.111±0.15^{abc}	1.516±0.55^{cd}	1.436±0.24^{bcd}	1.526±0.10^{bcd}	1.812±0.09^{a}	1.242±0.18^{b}	1.413±0.30^{ab}	1.553±0.08^{a}
**C123**	17.96±.77^{d}	17.6±2.01^{def}	14.05±2.73^{a}	12.23±0.75^{c}	12.34±0.74^{a}	1.57±0.61^{bc}	2.341±0.79^{abc}	2.346±0.72^{ab}	2.143±0.51^{a}	2.113±0.33^{a}	1.486±0.06^{cd}	1.493±0.16^{a}	1.746±0.10^{a}	1.143±0.15^{ab}	1.582±0.07^{a}
**C97**	19.70±1.33^{c}	16.08±0.78^{g}	11.74±0.39^{cd}	12.74±0.84^{bc}	8.623±0.78^{d}	1.88±0.31^{ab}	2.6767±0.15^{a}	2.506±0.19^{a}	1.866±0.59^{abc}	1.221±0.15^{cde}	1.741±0.10^{abc}	1.776±0.08^{a}	1.266±0.08^{b}	1.413±0.15^{ab}	1.387±0.06^{ab}
**C71**	21.97±1.03^{b}	20.22±0.32^{ab}	13.20±7.55^{ab}	13.77±0.97^{ab}	13.08±0.33^{a}	2.01±0.49^{ab}	2.213±0.52^{a-d}	2.443±0.09^{a}	1.916±0.17^{ab}	1.883±0.69^{ab}	1.792±0.12^{ab}	1.642±0.04^{a}	1.663±0.09^{a}	1.463±0.10^{a}	1.641±0.09^{a}
**C91**	21.25±1.66^{b}	17.36±1.16^{efg}	12.69±0.08^{bc}	11.77±1.78^{cd}	12.92±1.57^{a}	1.94±0.36^{ab}	1.953±0.64^{b-e}	2.136±0.43^{abc}	1.726±0.25^{bc}	1.712±0.37^{abc}	1.853±0.07^{a}	1.421±0.10^{a}	1.231±0.10^{b}	1.183±0.08^{ab}	1.281±0.13^{ab}

Data are presented as mean value with different letters which denote statistically significant difference between means within each indicator column among varieties according to Fisher's least significant difference (LSD) test. SFW: shoot fresh weight; RFW: root fresh weight; SL: shoot length; RL: root length and SDW: shoot dry weight.

Table S2. Effect of salt stress on No of leaves, leaf area, root surface area, and root volume of rapeseed seedlings in different varieties.

Variety	No. of leaves					Leaf area				
	Control	150 mM	200 mM	250 mM	300 mM	Control	150 mM	200 mM	250 mM	300 mM
**C280**	3.2±0.30^{bc}	2.8±0.11^{bcd}	2.8±0.12^{bc}	2.2±0.46^{cd}	1.8±0.23^{cd}	45.17±12.62^{d}	43.17±3.96^{a}	21.72±2.42^{de}	16.49±4.44^{g}	14.71±3.98^{f}
**C272**	5.4±0.23^{a}	4.4±0.64^{a}	4.4±0.23^{a}	3.4±0.11^{a}	2.2±0.34^{bc}	40.50±4.16^{e}	31.52±5.34^{c}	20.06±6.24^{e}	19.31±3.73^{f}	18.74±2.83^{d}
**C136**	3.8±0.11^{b}	2.6±0.23^{cd}	2.6±0.30^{c}	2.4±0.64^{bcd}	2.2±0.23^{bc}	44.34±17.67^{d}	30.45±3.46^{c}	30.25±1.89^{ab}	23.21±4.76^{d}	13.27±1.80^{g}
**C196**	3.4±0.11^{b}	2.4±0.30^{d}	2.4±0.11^{c}	2±0.12^{de}	2.2±0.2^{bc}	48.23±10.53^{c}	31.70±6.76^{bc}	22.91±2.05^{cd}	18.54±2.56^{f}	16.84±3.88^{e}
**C320**	3.4±0.20^{b}	2.2±0.30^{d}	2.4±0.52^{c}	1.6±0.30^{e}	1±0.14^{e}	41.31±4.93^{e}	33.59±1.48^{b}	24.14±0.86^{cd}	20.49±0.98^{e}	17.15±267^{e}
**C88**	3.4±0.26^{b}	3.2±0.11^{bc}	3±0.12^{bc}	2.6±0.11^{bc}	1.6±0.57^{d}	53.127±11.44^{a}	42.13±6.02^{a}	24.53±2.91^{c}	22.69±3.18^{d}	21.81±2.08^{c}
**C123**	3.4±0.23^{b}	3.4±0.4^{b}	2.6±0.11^{c}	2.6±0.11^{bc}	3.2±0.11^{a}	44.807±9.916^{d}	31.74±6.50^{bc}	30.76±3.91^{a}	25.82±1.89^{c}	30.24±4.17^{b}
**C97**	2.6±0.30^{c}	2.4±0.23^{d}	2.2±0.23^{c}	2.2±0.17^{cd}	2±0.10^{bcd}	49.193±10.96^{bc}	41.21±2.30^{a}	27.91±2.21^{b}	27.07±3.01^{b}	19.19±1.15^{d}
**C71**	3.4±0.2^{b}	2.4±0.52^{d}	2.6±0.64^{c}	2.4±0.11^{bcd}	2.4±0.20^{b}	50.367±18.63^{b}	42.16±8.60^{a}	29.79±3.52^{ab}	28.56±1.80^{a}	34.60±1.71^{a}
**C91**	3.4±0.30^{b}	2.8±0.30^{bcd}	3.6±0.30^{ab}	2.8±0.23^{b}	2.2±0.11^{bc}	49.723±8.50^{b}	42.88±1.89^{a}	24.89±5.67^{c}	20.52±4.75^{e}	13.76±4.55^{fg}
	**Root surface area**					**Root volume**				
**C280**	39.27±5.48^{cd}	19.68±1.07^{ab}	14.93±0.93^{ab}	12.77 ± 1.12^{bc}	7.07±0.61^{bc}	1.74±0.26^{bc}	0.97±0.21^{bcd}	0.61±0.07^{ab}	0.23±0.04^{d}	0.07±0.01^{f}
**C272**	41.70±2.49^{cd}	11.21±1.17^{e}	9.91±1.27^{d}	7.58 ± 0.83^{de}	5.32±0.74^{d}	1.88±0.16^{abc}	0.67±0.22^{de}	0.30±0.06^{c}	0.22±0.02^{d}	0.18±0.03^{cde}
**C136**	36.99±1.61^{cd}	17.24±1.06^{bc}	15.16±1.12^{a}	13.68 ± 0.5^{b}	8.36±0.84^{ab}	1.77±0.29^{abc}	1.04±0.12^{abc}	0.36±0.28^{bc}	0.27±0.06^{cd}	0.25±0.03^{abc}
**C196**	40.78±1.34^{cd}	16.13±0.9^{cd}	12.93±0.94^{abc}	12.63 ± 1.25^{bc}	9.88±1.21^{a}	1.84±0.14^{abc}	1.22±0.21^{ab}	0.60±0.27^{ab}	0.42±0.16^{c}	0.28±0.03^{ab}
**C320**	38.06±1.91^{cd}	15.80±0.69^{cd}	11.28±2.04^{cd}	6.91 ± 1.03^{e}	5.38±0.89^{d}	1.89±0.19^{abc}	0.54±0.07^{e}	0.30±0.02^{c}	0.23±0.05^{c}	0.21±0.04^{bcd}
**C88**	51.45±5.56^{ab}	16.71±1.57^{cd}	10.33±1.25^{d}	9.23 ± 1.23^{d}	5.60±1.02^{cd}	2.32±0.57^{a}	1.12±0.16^{abc}	0.55±0.04^{bc}	0.40±0.11^{c}	0.17±0.01^{de}
**C123**	36.53±0.79^{d}	16.20±0.79^{cd}	12.04±1.36^{cd}	11.33 ± 1.32^{c}	6.00±0.87^{cd}	1.96±0.42^{ab}	0.45±0.07^{e}	0.60±0.09^{ab}	0.27±0.06^{cd}	0.11±0.02^{ef}
**C97**	46.33±5.15^{bc}	15.69±1.6^{cd}	12.73±0.62^{bc}	9.15 ± 1.52^{d}	5.53±1.02^{cd}	1.81±0.32^{abc}	0.88±0.14^{cd}	0.51±0.11^{bc}	0.72±0.12^{ab}	0.30±0.08^{a}
**C71**	59.45±12.84^{a}	20.11±3.01^{ab}	15.21±1.10^{a}	15.99 ± 1.24^{a}	7.91±1.34^{ab}	2.24±0.41^{ab}	1.33±0.29^{a}	0.86±0.22^{a}	0.75±0.09^{a}	0.22±0.04^{bc}
**C91**	45.53±6.04^{bcd}	14.60±1.71^{d}	10.77±2.21^{cd}	8.37 ± 1.12d^{e}	5.61±0.44^{cd}	1.44±0.31^{c}	0.92±0.12^{cd}	0.59±0.07^{b}	0.60±0.11^{b}	0.23±0.08^{a-d}

Data are presented as mean value with different letters which denote statistically significant difference between means within each indicator column among varieties according to Fisher's least significant difference (LSD) test.

Table S3. Effect of salt stress on physiological traits of rapeseed seedlings in different varieties.

Variety	Total chlorophyll					Total amino acid					TSS				
	**Control**	**150 mM**	**200 mM**	**250 mM**	**300 mM**	**Control**	**150 mM**	**200 mM**	**250 mM**	**300 mM**	**Control**	**150 mM**	**200 mM**	**250 mM**	**300 mM**
**C280**	1.269±0.122^{bc}	1.055±0.08^{bcd}	0.348±0.14^{e}	0.482±0.06^{c}	0.380±0.14^{d}	9.853±2.45^{c}	15.63±2.38^{b}	21.59±0.64^{a}	22.44±3.92^{a}	23.78±1.57^{b}	11.89±1.19^{c}	20.85±0.97^{cd}	22.00±2.47^{cd}	23.85±3.13^{cd}	20.77±1.65^{b}
**C272**	1.133±0.04^{cde}	0.895±0.06^{e}	0.249±0.01^{e}	0.794±008^{b}	0.336±0.02^{d}	10.58±0.87^{bc}	13.68±0.74^{c}	18.53±1.99^{b}	19.32±1.87^{b}	16.35±3.91^{c}	15.67±0.51^{b}	38.19±3.88^{a}	36.82±2.33^{a}	35.85±4.14^{a}	22.66±3.40^{b}
**C136**	1.094±0.02^{de}	0.991±0.13^{cde}	0.656±0.08^{d}	0.657±0.02^{bc}	0.295±0.03^{d}	14.86±3.89^{b}	16.47±2.63^{ab}	19.20±3.24^{a}	19.36±1.51^{b}	24.87±9.66^{b}	19.58±0.73^{a}	22.51±0.81^{c}	24.01±1.27^{bc}	19.87±1.46^{def}	12.50±1.34^{cd}
**C196**	1.463±0.21^{a}	1.118±0.08^{bc}	1.011±0.15^{b}	0.619±0.12^{bc}	0.605±0.02^{bc}	9.463±2.45^{c}	10.67±0.96^{c}	16.643±10.8^{c}	21.66±19.65^{a}	26.19±12.8^{ab}	17.03±0.80^{b}	26.47±6.70^{b}	21.89±1.51^{cd}	19.44±4.93^{ef}	10.22±1.63^{d}
**C320**	0.967±0.04^{ef}	0.995±0.12^{cde}	1.065±0.14^{ab}	0.569±0.17^{c}	0.802±0.07^{a}	9.856±2.54^{c}	16.07±3.41^{ab}	18.48±2.92^{b}	21.48±0.98^{a}	23.14±6.03^{b}	19.29±0.94^{a}	23.31±1.91^{d}	26.10±0.55^{b}	27.04±1.94^{bc}	22.93±2.16^{ab}
**C88**	1.219±0.10^{bc}d	1.116±0.03^{bc}	0.89±0.018^{bc}	0.492±0.08^{c}	0.269±0.03^{d}	13.33±2.34^{bc}	15.24±2.93^{b}	17.21±2.72^{c}	23.54±12.90^{a}	26.37±6.28^{ab}	9.766±0.69^{d}	15.21±1.26^{f}	19.93±10.7^{d}	18.16±1.69^{f}	15.24±2.06^{c}
**C123**	1.312±0.049^{ab}	1.167±0.05^{b}	0.407±0.05^{e}	0.662±0.02^{bc}	0.804±0.08^{a}	10.87±2.13^{bc}	13.13±2.45^{c}	16.58±1.79^{c}	18.70±1.43^{b}	10.93±3.88^{c}	7.703±0.76^{d}	16.09±2.11^{ef}	19.07±0.48^{d}	22.40±2.59^{de}	23.91±0.95^{ab}
**C97**	1.296±0.02abc	1.094±0.03^{bcd}	0.993±0.07^{b}	0.786±0.18b	0.612±0.10^{bc}	15.06±1.54^{a}	18.63±3.62^{a}	20.20±2.91^{a}	20.68±1.93^{a}	28.68±14.29^{a}	16.06±1.27^{b}	23.46±1.03^{bc}	18.65±2.69^{d}	13.37±0.75^{g}	21.18±2.39^{b}
**C71**	1.261±0.15^{f}	0.971±0.02^{de}	0.728±0.13^{cd}	0.983±0.11^{a}	0.720±0.06^{ab}	9.593±1.50^{c}	18.02±7.74^{a}	19.40±1.56^{a}	23.15±1.69^{a}	30.88±8.47^{a}	19.54±1.14^{a}	22.94±1.71^{c}	26.03±2.25^{b}	28.26±1.02^{b}	26.86±2.53^{a}
**C91**	1.198±0.05^{bcd}	1.495±0.03^{a}	1.231±0.08^{a}	0.648±0.03^{bc}	0.526±0.01^{c}	10.18±3.36^{c}	16.73±1.92^{ab}	19.70±3.67^{a}	15.96±10.7^{c}	26.73±4.14^{ab}	16.51±0.74^{b}	19.07±1.82^{ef}	21.52±1.79^{cd}	22.61±3.81^{de}	9.606±0.53^{d}
	**TSP**					**Proline**					**MDA**				
**C280**	117.1±7.02^{b}	121.3±0.55^{b}	109.2±3.21^{c}	121.7±1.94^{b}	115.0±4.23^{b}	1.349±0.21^{a}	3.352±0.11^{ab}	3.697±0.07^{bc}	3.723±0.06^{b}	3.611±0.08^{b}	0.497±0.01^{ab}	0.589±0.05^{a}	0.628±0.06^{a}	0.659±0.01^{a}	0.681±0.07^{ab}
**C272**	121.6±2.22^{ab}	128.3±5.18^{ab}	124.6±2.51^{ab}	122.6±2.26^{b}	128.7±12.5^{ab}	1.790±1.55^{bcd}	3.447±0.11^{ab}	3.709±0.07^{bc}	3.762±0.05^{b}	3.747±0.06^{b}	0.411±0.01^{b}	0.472±0.04^{c}	0.550±0.03^{ab}	0.543±0.02^{ab}	0.585±0.02^{bcd}
**C136**	127.6±4.47^{a}	129.1±8.23^{ab}	113.9±1.15^{bc}	127.3±22.5^{b}	128.0±9.61^{ab}	2.483±0.02^{abc}	3.106±0.06^{a}	3.569±0.06^{bc}	3.489±0.09^{bc}	3.382±0.10^{c}	0.479±0.05^{ab}	0.522±0.05^{bc}	0.547±0.05^{ab}	0.574±0.01^{ab}	0.586±0.01^{bcd}
**C196**	127.2±3.19^{a}	130.2±1.50^{a}	132.6±3.31^{a}	125.3±7.42^{b}	109.5±3.68^{b}	2.701±0.11^{ab}	3.334±0.08^{b}	3.731±0.04^{bc}	4.045±0.05^{ab}	4.498±0.09^{b}	0.429±0.02^{b}	0.497±0.04^{c}	0.537±0.11^{ab}	0.564±0.01^{ab}	0.583±0.03^{bcd}
**C320**	123.2±4.06^{ab}	129.3±2.62^{ab}	102.8±12.02^{c}	106.6±5.06^{c}	104.5±16.7^{b}	2.182±0.19^{a-d}	3.501±0.01^{a}	3.512±0.11^{bc}	3.706±0.09^{b}	3.629±0.16^{b}	0.413±0.03^{b}	0.464±0.01^{c}	0.547±0.04^{ab}	0.562±0.01^{ab}	0.575±0.01^{cd}
**C88**	123.8±2.93^{ab}	125.7±4.13^{ab}	111.9±3.71^{bc}	112.9±1.91^{bc}	122.3±1.29^{ab}	2.688±0.22^{ab}	3.550±0.04^{a}	3.471±0.07^{c}	3.386±0.08^{c}	3.348±0.13^{c}	0.487±0.01^{ab}	0.522±0.03^{bc}	0.582±0.01^{ab}	0.608±0.11^{a}	0.699±0.01^{a}
**C123**	123.0±2.44^{ab}	127.3±3.31^{ab}	129.1±3.36^{a}	130.2±8.41^{b}	131.1±3.75^{ab}	2.858±1.58^{a}	3.492±0.07^{a}	4.131±0.07^{ab}	4.159±0.09^{a}	4.401±0.017^{b}	0.527±0.01^{a}	0.589±0.01^{a}	0.593±0.01^{ab}	0.596±0.01^{ab}	0.576±0.03^{cd}
**C97**	117.9±2.23^{b}	122.0±3.06^{ab}	113.5±3.34^{bc}	126.7±0.68^{b}	117.0±6.14^{b}	1.633±0.03^{cd}	3.528±0.02^{a}	3.966±0.07^{abc}	3.883±0.06^{b}	3.760±0.06^{b}	0.425±0.28^{b}	0.483±0.07^{c}	0.501±0.06^{b}	0.480±0.02^{b}	0.501±0.05^{d}
**C71**	119.2±2.98^{b}	129.2±5.63^{ab}	140.4±4.08^{a}	162.4±1.94^{a}	172.8±6.02^{a}	1.487±0.26^{c}	3.517±0.09^{a}	4.387±0.08^{a}	4.889±0.13^{a}	5.352±0.08^{a}	0.525±0.06^{a}	0.465±0.09^{c}	0.535±0.06^{b}	0.530±0.01^{b}	0.573±0.01^{bcd}
**C91**	117.6±4.87^{b}	122.1±7.95^{ab}	120.3±6.24^{b}	151.1±40.9^{ab}	132.7±23.6^{ab}	1.523±0.88^{cd}	3.607±0.02^{a}	3.761±0.11^{abc}	3.525±0.02^{bc}	3.560±0.01^{b}	0.470±0.01^{ab}	0.577±0.02^{b}	0.603±0.09^{a}	0.56±0.02^{b}	0.651±0.02^{abc}

Data are presented as mean value with different letters which denote statistically significant difference between means within each indicator column among varieties according to Fisher's least significant difference (LSD) test. TSS: total soluble sugar; TSP: total soluble protein and MDA: malonaldehyde.
